# Joint stochastic optimisation of stope layout, production scheduling and access network

**DOI:** 10.1177/25726668241242230

**Published:** 2024-04-13

**Authors:** Cristina Penadillo, Roussos Dimitrakopoulos, Mustafa Kumral

**Affiliations:** 1 COSMO – Stochastic Mine Planning Laboratory, 5620 McGill University, Montreal, Canada; 2 Department of Mining and Materials Engineering, 5620 McGill University, Montreal, Canada

**Keywords:** Stochastic underground mine optimisation, sublevel stoping, stope design, production scheduling, access networks, dilution management

## Abstract

The three main optimisation components of sublevel stoping methods are stope layout, production schedule (or stope sequencing) and access networks. The joint optimisation of these components could further add value to an underground mining project. This potential has not been considered in the literature due to computational difficulties, and the problem was solved sequentially. This paper proposes a new joint optimisation model to integrate these components. In addition, the proposed optimisation model incorporates stochastic simulations to capture uncertainty and variability associated with the grades of the related mineral deposits mined. The optimisation model is based on a two-stage stochastic integer programming (SIP) formulation that maximises the project's net present value (NPV) and minimises the planned dilution. Applying the proposed method at a small copper deposit shows that the SIP outperforms the results obtained from mixed integer programming. For a seven-year mine life, the SIP model generated ∼20% more NPV, demonstrating the importance of developing a joint optimisation formulation and accounting for grade uncertainty and variability.

## Introduction

Open-pit mine planning (OPMP) research has significantly progressed over the last two decades. In line with this progress, OPMP software products have evolved. Mature, more accurate, and fast mine planning tools have made open-pit operations more profitable and sustainable. However, underground mine planning (UMP) continues to grow, and robust software tools remain relatively limited. There are three reasons why UMP tools lag relatively behind OPMP tools: (a) UMP is more computationally complex; for example, in the optimisation process, checking whether a block belongs to a stope requires a large number of iterations. (b) Underground mining methods are diverse, and UMP tools cannot be generalised for all methods. Planning tools must be tailored to the specific characteristics of each method. (c) Rock mechanics and stability constraints are paramount. Therefore, the value-added potential of UMP is limited compared to OPMP; that is, mine design aspects restrict manoeuvring room for mine planning optimisation (Sari and Kumral, 2021).

Engineering optimisation processes sometimes attempt to solve large problems. However, computational resources may not have sufficient capacity to solve them. Thus, these problems are divided into relatively smaller subproblems, whereby each is solved independently. In this process, however, the sum of the optimal solutions of sub-problems cannot guarantee the creation of an optimal solution to a combined large optimisation problem. In addition, dividing a problem into sub-problems can pose a paradoxical issue. Each sub-problem requires the solution of the other sub-problem as input. Unless some parameters are assumed, the sub-problems cannot be solved. The solution to the sub-problems can be mathematically optimal, but since some parameters are assumed, there could be significant deviations from the actual project value. Therefore, optimising sub-problems does not guarantee the optimality of solutions for large, combined problems, as it does not consider jointly the interaction and influence between all components throughout the mining process (Hou et al., 2019a; Kumral, 2013; Little et al., 2013).

The abovementioned paradoxical issue occurs in the UMP problem. The access network optimisation focuses on determining permanent shaft or decline connecting to production stopes. Material handling costs are required as input in stope layout (boundary) optimisation (SLO). If the access point and network to the mine are unknown, material handling costs cannot be estimated. Regardless of how advanced software or formulation is for the SLO, the project may lose value due to the assumption made for materials handling costs. On the other hand, if the locations of production stopes are unknown, it is impossible to determine the location of mine access. This reasoning can be applied to all sub-problem pairs.

The mining method studied in this paper is sublevel stoping (SLS), which is one of the most used methods for large-scale vertical deposits. The requirement of this method is that the footwall dip of the orebody must be greater than the angle of repose of the broken ore, with a regular shape and competent host rock. SLS involves accessing the ore through sublevels located between the main haulage levels. Production holes are drilled in rings parallel to the dip of the orebody between the drilling drives (sublevels). Next, the mining sequence is given by blasting the production rings into the advancing void; the broken material is recovered from the extraction or drawpoints (levels) (Basiri, 2018; Dowd and Elvan, 1987; Villaescusa, 2014). The resulting voids generated by the extraction of stopes are usually backfilled, where the backfill material provides support and confinement for further mining of the surrounding stopes (Little et al., 2008, 2011, 2013).

UMP for SLS aims to find the stopes along with their extraction periods and access network that maximises the net present value (NPV) of an operation. This UMP problem is divided into three components: (i) SLO, (ii) production scheduling (or sequencing), and (iii) access network optimisation. The stope layout refers to the search of all possible combinations of blocks, delineated by the geometric, geotechnical, and operational constraints, which maximise the profit of the mine while considering the related spatial locations, such as the positioning of the sublevels and levels (Faria et al., 2021; Hou et al., 2019b). Production scheduling refers to the sequencing of the stopes that maximises the discounted cash flows of the mine and considers stability, development activities, equipment transportation, and capacities. This sequencing is based on the information obtained from the stope layout and the available access networks (Nehring et al., 2010; Sari and Kumral, 2021; Villalba and Kumral, 2019). Access roads and networks refer to the transport system for the mined material; it is a set of interconnected shafts or declines, drives, ramps, and drifts (sometimes including ore passes) that serve to transport ore material to the mill (Brazil and Grossman, 2008) and, if waste material is present, the latter is transported to the nearest waste pile for subsequent use as backfill material. The construction costs of the infrastructure (e.g. shaft or decline) are considered an important portion of the capital expenditures of the project, and the access roads connecting the infrastructure to the stopes (called preparations) are considered a part of the operational expenditures. The inclusion of the latter component has the function of minimising the associated development and haulage costs (Hou et al., 2019a).

Some studies have been conducted to solve each sub-problem separately. In stope layout, Alford (1995), Alford et al. (1999), Ataee-Pour (2004), Alford et al. (2007), and Bai et al. (2013) implemented mixed integer programming (MIP) models; Topal and Sens (2010), Sandanayake et al. (2015), and Sari and Kumral (2021) use heuristic methods. However, these approaches do not deal with grade uncertainty. Some relevant stochastic models that include geological grade uncertainty are found in Dimitrakopoulos and Grieco (2009), Villalba and Kumral (2019), and Faria et al. (2021, 2022). MIP formulations have been proposed to generate production schedules that maximise the discounted cash flows (Carlyle and Eaves, 2001; King et al., 2017; Kuchta et al., 2004; Little et al., 2008; Nehring and Topal, 2007; Nehring et al., 2010, 2013; Newman et al., 2007; O'Sullivan and Newman, 2014; Ogunmodede et al., 2022; Sarin and West-Hansen, 2005; Smith et al., 2003; Terblanche and Bley, 2015; Topal, 2003; Trout, 1995), adding a heuristic approach by Fava et al. (2013). Some stochastic methods that deal with geological uncertainty were developed by Carpentier et al. (2016), Huang et al. (2020), and Nesbitt et al. (2021). In terms of access roads, the system of connected networks to access the stopes and transport the ore material to the mill at the surface is studied by Brazil and Thomas (2007), Brazil and Grossman (2008), and Yardimci and Karpuz (2019). The goal of these approaches is to generate a network layout that minimises costs for accessing points at each production level.

Some further studies have been carried out to optimise jointly the processes and avoid addressing each sub-problem independently. Integrated deterministic approaches for stope layout and production scheduling are found in Basiri (2018), Copland and Nehring (2016), Foroughi et al. (2019), Little et al. (2011, 2013), and Nehring et al. (2010, 2013), while a stochastic implementation is discussed in Faria et al. (2022). A study on the integration of stope layout and access networks, which implements a non-linear mixed integer program, is found in Hou et al. (2019b).

Regarding the integration of stope layout and access networks as a function of time, Hou et al. (2019b) proposed an integer programming (IP) formulation; however, this approach is different from the model presented in this paper. Their approach is limited to a single shaft option, as well as predefined production levels and access drives. Their model attempts to find out when the predesigned path is activated, rather than which access option is chosen. Furthermore, their method does not address geomechanical controls, which are an important factor in determining whether or not a long-term plan is viable. Also, their model does not consider the set of simulations simultaneously, evaluating each geological model independently and then choosing the mining plan that results in the maximum NPV.

Stochastic approaches incorporate grade uncertainty and variability into the optimisation process through a set of equally probable simulations of the mineral deposit, overcoming unrealistic scheduling designs (Carpentier et al., 2016; Dimitrakopoulos et al., 2002; Dimitrakopoulos and Grieco, 2009; Dowd and Elvan, 1987; Faria et al., 2022; Godoy, 2002; Hou et al., 2019b; Huang et al., 2020; Jewbali, 2015; Myers et al., 2007; Ramazan and Dimitrakopoulos, 2013; Rendu, 2017; Vallée, 2000; Villalba and Kumral, 2019). This paper presents a two-stage stochastic integer programming (SIP) (Birge and Louveaux, 1997) that integrates the three components while managing planned dilution and incorporating grade uncertainty with 10 stochastic simulations. Stopes are composed of rings, which are blasted in a controlled sequence so that the rings are sent to two different destinations: ore to the mill at the surface and waste to the temporary waste piles at the extraction levels. The purpose of planned dilution management is to ensure the maximum mining recovery of material with the maximum economic value, which translates into minimal ore planned dilution, lower costs, and minimal ore losses (Villalba and Kumral, 2017). Scheduled stopes should be fully mined, but, in many cases, planned dilution represented by rings with negative economic values are unavoidable. Moreover, in order to avoid ruling out stope candidates, it is important to give flexibility to the optimiser to schedule stopes and make decisions about their rings to minimise the inherent internal dilution within the stopes. A set of infrastructure options is provided for the optimiser to determine the optimal scheduled stope layout and access network that maximises the NPV of the project and minimises the associated costs.

The following section shows the methods used to develop the joint optimisation formulation. Then, the performance of the proposed methodology is demonstrated by a case study. Finally, the conclusions will be provided, and potential extensions for future research will be given.

## Method

This research proposes a stochastic mathematical programming model to maximise the NPV of an underground mining operation, based on the sub-level stoping method. It integrates three related mine planning sub-problems, namely: stope layout, production scheduling, and access network.

In the model, the main input parameter is small blocks. When a certain number of small blocks are combined, rings are formed. The rings are combined to create stopes. Working with blocks, rings, and stopes facilitates dilution management. In the proposed model, stopes are not assessed as ore as a whole. Waste rings are sent to waste dumps through controlled blasting. Also, this treatment helps mine planners reproduce grade variability. The NPV maximization model schedules the stopes and selects the rings that are sent to the processing mill by accounting for the geological uncertainty given by the set of stochastic orebody simulations s∈S . The SIP evaluates stope layouts with variable stope lengths, where a stope i∈I is composed of rings r∈R , and each ring is composed of blocks b∈B . The maximum and minimum allowed dimensions for a stope are defined by the geomechanical and operational conditions of the mine, as well as by the geometry of the deposit. For simplicity, an assumption is that the rings considered as waste are not transported to the surface and are piled in the nearest level for backfilling usage.

**Figure 1.**
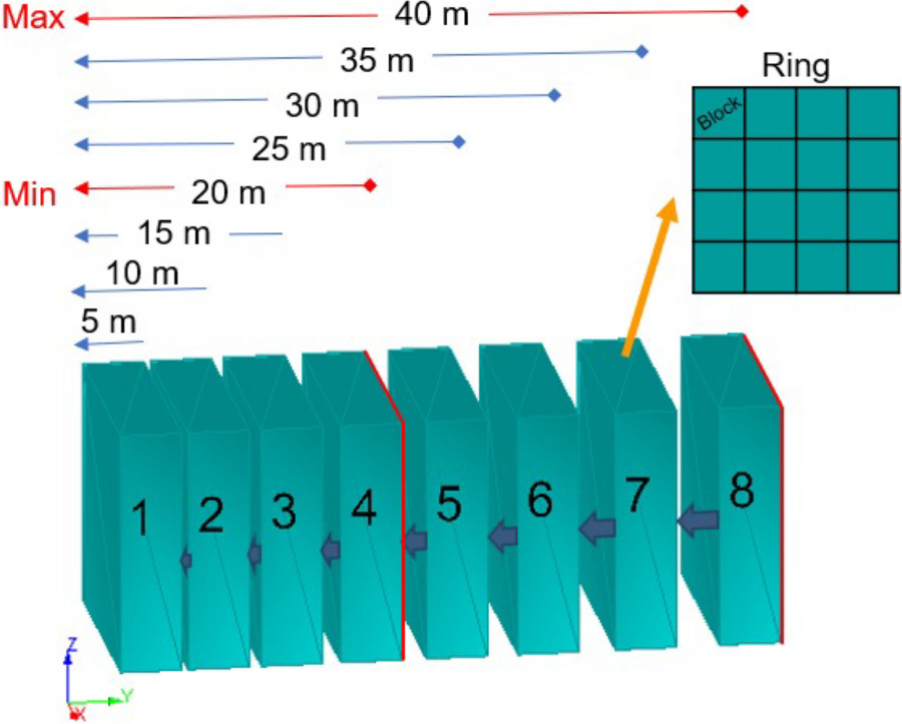
Stope showing a ring of 4 × 4 blocks and a stope of minimum 4 rings to a maximum of 8 rings.

Figure 1 presents a stope composed of eight rings, where each ring is composed of 4 × 4 blocks and the dimension of each block is 5 × 5 × 5 m. The proposed approach allows a minimum and maximum length of the stopes to evaluate the different stope shapes in that interval, which means that there is no predetermined stope shape in the model. The stope in the figure has minimum and maximum allowable lengths of 20 and 40 m, respectively, forming a total of five stope combinations, composed of 4, 5, 6, 7, or 8 rings.

**Figure 2.**
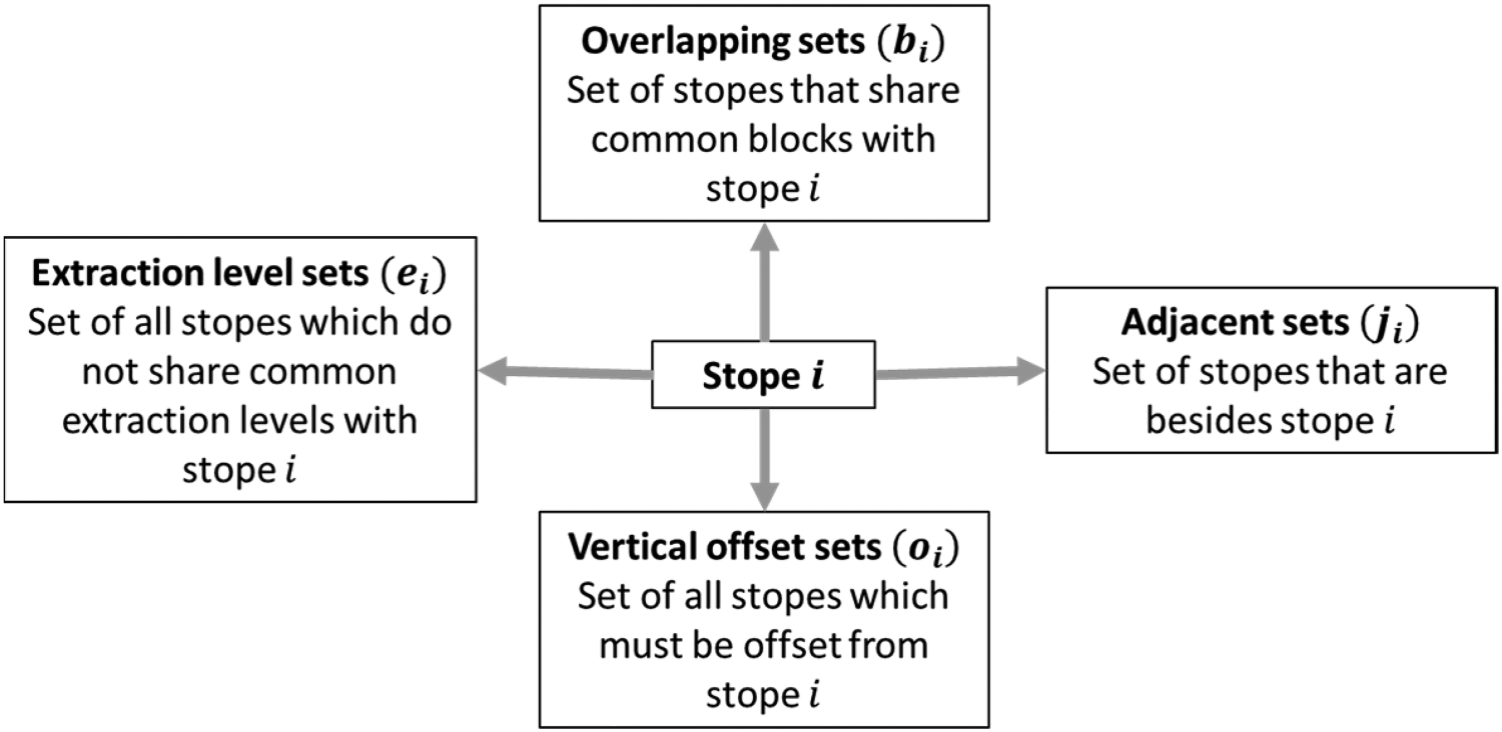
Process of generating operable, stable, and accessible stopes.

Operability, stability, and adequate accessibility constraints must be met. Sets, such as overlapping $\left(b_{i}\right)$ , adjacent $\left(\;j_{i}\right)$ , vertical offset $\left(o_{i}\right)$ , , and extraction level $\left(e_{i}\right)$ , are identified for each stope i∈I (Figure 2). The R-tree data structure (Guttman, 1984) is implemented to reduce the search time of these sets. This spatial search method identifies the group of nearby stopes bounded within the limits of a defined area. In this case, a three-dimensional search is performed by searching on the *x*, *y*, and *z* axes from the evaluated stope *i* delimited in a neighbourhood search of up to two stopes distance.

**Figure 3.**
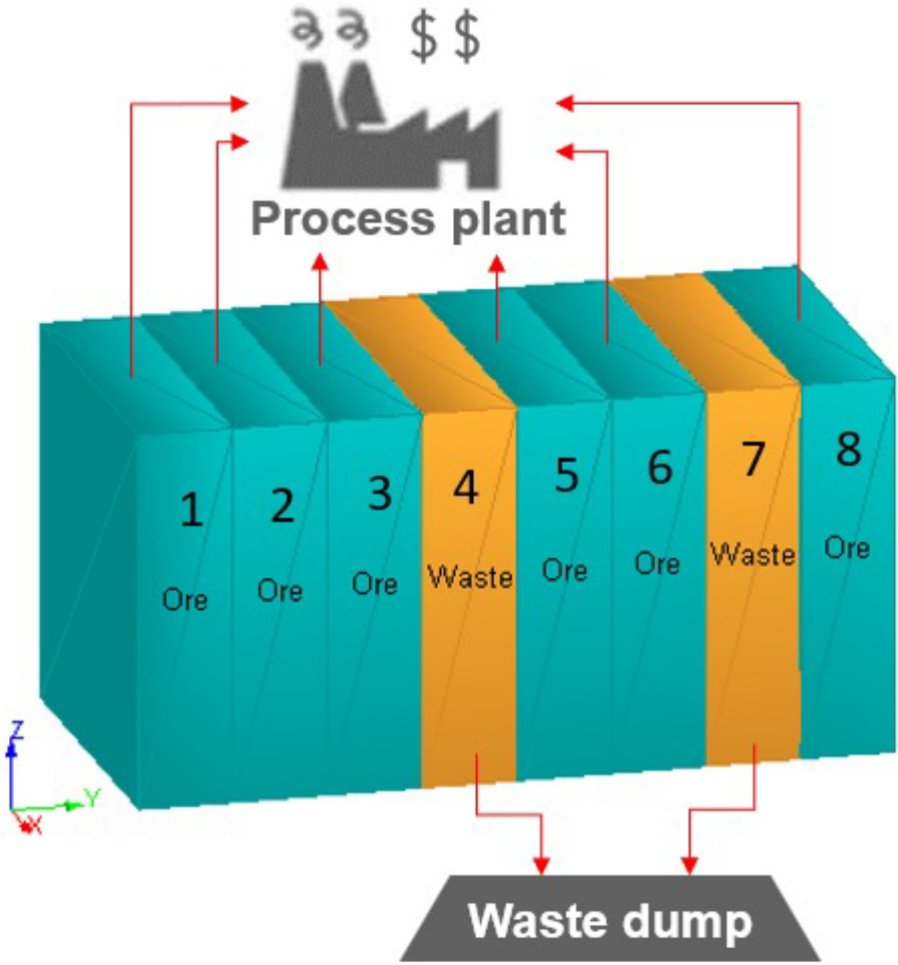
Illustration of internal dilution within a stope.

Figure 3 illustrates a mining stope containing eight rings where six rings are ore, and two rings are waste. Each ring is identified as ore or waste, applying a cut-off grade. A stope is formed by combining rings. Working with rings allows engineers to control internal dilution. The optimisation model described below generates stopes combining rings under operability and geomechanical constraints to maximise profit. As much as possible, the optimisation formulation forces to be incorporated ore rings into stopes and waste rings to be outside of stopes. Furthermore, this formulation informs blasting patterns. Controlling blasting can also help ore and waste separation. Thus, internal dilution is minimised. Operability refers to the open face in the blasting sequence of the rings; for example, in Figure 3, rings 4 and 7 are defined as waste rings, but for blasting rings 5, 6, and 8, they need an open face, so rings 4 and 7 must be blasted. The geomechaincal constraints are described through the period required for curing and leaving waste rings outside of stopes as possible. In the model, two adjacent stopes cannot be produced in two consecutive periods due to stability requirements. When a stope is produced, the adjacent stope can be produced after the waiting time required for curing. This time is given by the rock mechanics engineer. Alternatively, if a certain number of rings is left as pillars, the curing time requirement can be overridden. The required number of pillars and curing time is specified in the optimisation formulation.

Access networks are considered by defining a set of access designs. The proposed model process would attempt to jointly optimise the stope layout, production schedule, and decline/shaft option that generates the most benefits. The number of access options is limited by the information based on the geomechanics engineer's calculations. The only requirement is that the designs must have input values of access *x*, *y*, and *z* coordinates and lengths at each level.

**Figure 4.**
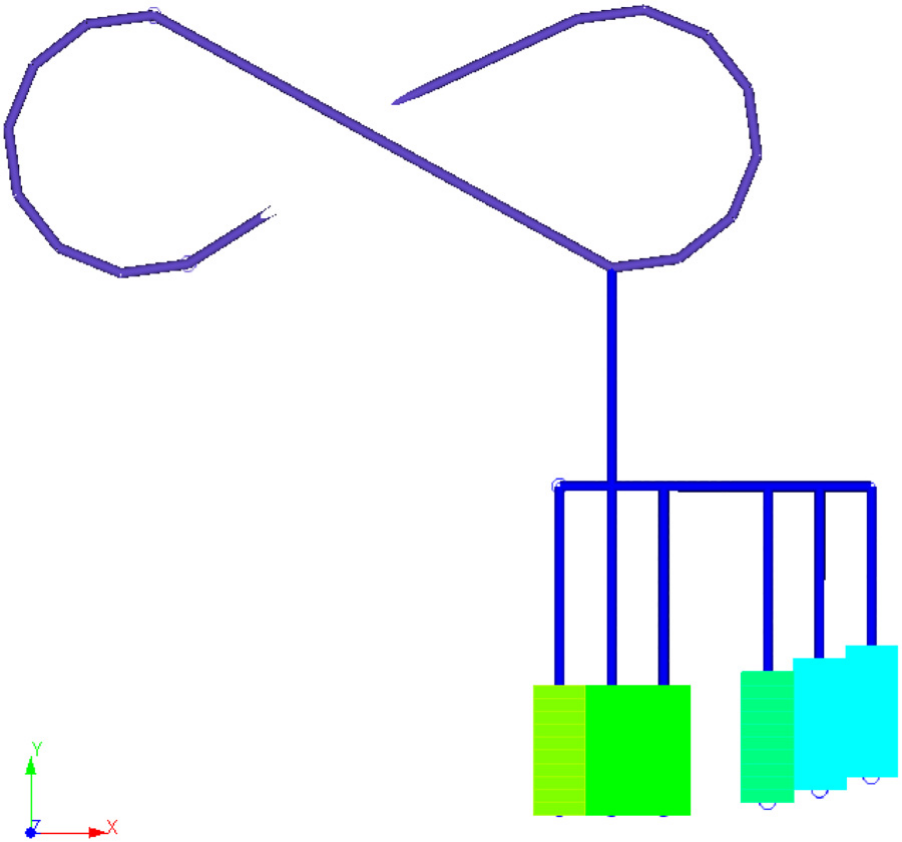
Illustration of decline access to stopes at one level (plan view).

Traditional stope optimising approaches ignore the effect of materials handling costs associated with access networks. The distance of a stope to the main access roads is an important parameter. The closeness to the shaft, decline, or primary crusher makes a difference for optimisation because the value of a stope depends on the materials handling costs, as well as the stope's grade. This issue is shown in Figure 4. This figure illustrates that each stope has a different distance to the decline. For example, the distance between the decline and stopes shown in green is shorter than the distance between the decline and the stopes shown in blue. Therefore, their material handling costs cannot be the same. To calculate these costs, we need to know the haulage network design earlier. On the other hand, if we do not know the locations of production stopes, we cannot design the access network. These distances are calculated as a city block distance. The city block distance (or Manhattan distance) is a technique that calculates the path between two objects. The city block distance technique is used for calculating the distance between two locations for given coordinates *x* and *y* at the same level *z* in the problem described in the paper.

**Figure 5.**
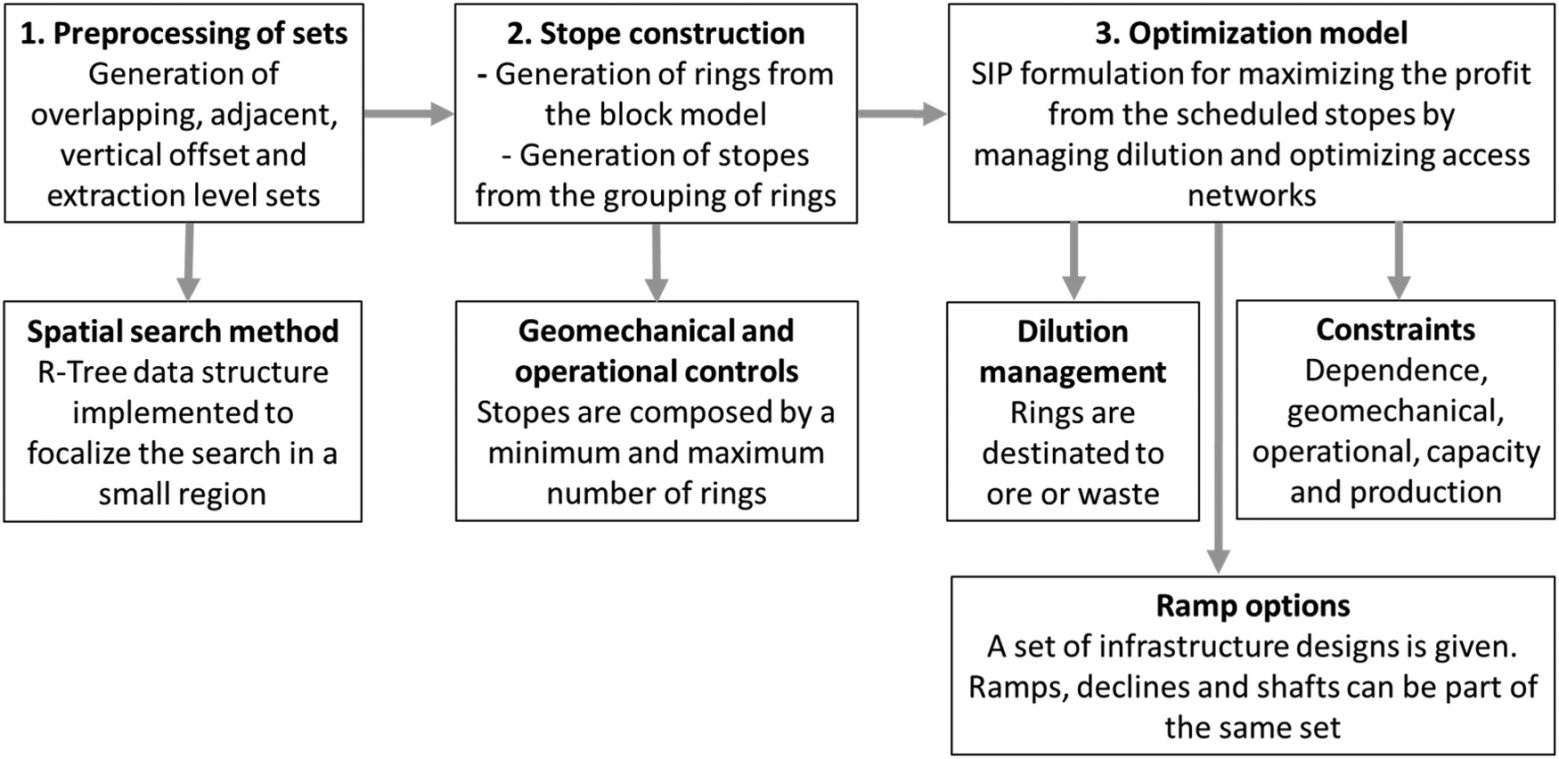
The workflow of the proposed optimisation process.

The steps of the proposed optimisation process are shown in Figure 5. The first step is to generate a feasible stope set. It follows the stope construction. At this step, different stope sizes are defined by the accumulation of rings, delimited by the minimum and maximum lengths informed by the stability assessment outcome. The last step is the optimisation process, where the joint process of generating a production schedule, stope layout, and accessing the network is executed. The mathematical model maximises NPV generated from an underground mining operation based on sublevel stopping. It also selects an access option that minimises the costs of development and preparations, which allows for the optimal extraction flow of the extracted material. Dependency between a stope and its rings is a constraint governing that all rings within a stope must mined. Geomechanics constraint controls if the maximum space size for stopes is exceeded. Operability defines whether stopes are accessible from level to level. Capacity and production constraints define the minimum and maximum targets of tonnage and metal content sent to the mill.

### Decision variables

Three decision variables are defined. The first variable decides if a stope is scheduled for production, the second variable determines the destination of the rings, and the third variable decides what decline option is selected.

$$x_{iot}=\left\{ \begin{array}{cc} 1,\; & \mathrm{stope}\;i\;\mathrm{is}\;\mathrm{mined}\;\mathrm{in}\;\mathrm{period}\;t\;\mathrm{for}\;\mathrm{decline}\;\mathrm{option}\;o\\ 0, & \mathrm{otherwise} \end{array}\right.$$

$$y_{rodt}=\{\begin{array}{cc} 1, & \mathrm{ring}\;r\;\mathrm{is}\;\mathrm{sent}\;\mathrm{to}\;\mathrm{destination}\;d\;\mathrm{in}\;\mathrm{period}\;\\ & t\;\mathrm{through}\;\mathrm{decline}\;\mathrm{option}\;o\\ 0, & \mathrm{otherwise} \end{array}$$

$$z_{o}=\{\begin{array}{cc} 1, & \mathrm{decline}\;\mathrm{option}\;o\;\mathrm{is}\;\mathrm{activated}\\ 0, & \mathrm{otherwise} \end{array}$$

Since $x_{iot}$ and $y_{rodt}$ are of binary type per each period *t*, a stope and a ring are fully mined at only one period *t*. Partially mining of stopes and rings is not considered for this study.

Additional continuous recourse variables are used to balance positive and negative deviations from meeting the grade targets, in period *t*, and scenario *s*.

$$\delta_{ts}^{g+},\delta_{ts}^{g-}\geq 0$$

### Mathematical formulation


(1) $$\begin{array}{r} \mathrm{Max}\;\;\frac{1}{S}\underset{\mathrm{Part}\;1}{\underbrace{\underset{r\in R}{\sum }\;\underset{o\in O}{\sum }\;\underset{d\in D}{\sum }\;\underset{t\in T}{\sum }\;\underset{s\in S}{\sum }}}\;\;\propto_{rodts}y_{rodt}-\underset{\mathrm{Part}\;2}{\underbrace{\underset{i\in I}{\sum }\;\underset{o\in O}{\sum }\;\underset{t\in T}{\sum }\;\beta_{iot}x_{iot}}}\;-\frac{1}{S}\underset{\mathrm{Part}\;3}{\underbrace{\underset{t\in T}{\sum }\;\underset{s\in S}{\sum }\;c_{t}^{g+}\delta_{ts}^{g+}+c_{t}^{g-}\delta_{ts}^{g-}}}-\underset{\mathrm{Part}\;4}{\underbrace{\underset{o\in O}{\sum }\;fl_{o}z_{o}}} \end{array}$$

The objective function of the SIP is composed of four parts. Part 1 aims to maximise the discounted cash flow of the processed rings. Part 2 minimises the extraction costs for mining the stopes. Part 3 is geologic risk management, which minimises the costs of deviating from planned production targets. While Part 4 represents the capital cost for the chosen decline option. Part 3 is incorporated for deviations associated with grade requirements, where the penalty costs $c^{g+}\mathrm{and}\;c^{g-}$ are defined according to the testing of different orders of magnitude and are discounted on time by a geological discount factor $dr^{\mathrm{geo}}$ (Benndorf and Dimitrakopoulos, 2013; Ramazan and Dimitrakopoulos, 2004, 2013).

The reserve constraints ensure that a stope *i* or a ring *r* is mined at most once during the entire life of mine *T*. Equation (5) is a reserve constraint for stopes, and equation (6) is for rings. The latter ensures that a ring activates one decline option *o*, and it is fully mined at most once along all periods.

(2) $$\begin{array}{c} \underset{o\in O}{\sum }\;\underset{t\in T}{\sum }\;x_{iot}\leq 1\;\forall \;i\in I \end{array}$$

(3) $$\begin{array}{c} \underset{d\in D}{\sum }\;\underset{t\in T}{\sum }\;y_{ordt}\leq z_{o}\;\forall \;r\in R,\;o\in O \end{array}$$

Equation (7) guarantees that only one access option is selected from a set of options (declines, etc.). For example, in the case study, seven possible decline options are given, and this constraint ensures that one of these options is only selected in the optimisation process.

(4) $$\begin{array}{c} \underset{o\in O}{\sum }\;z_{o}\leq 1 \end{array}$$

The dependency between stope *i* and ring *r* is ensured in equations (8) and (9). In the first one, a ring can be mined only if the stope to which it belongs is mined. Equation (9) ensures that, among all stopes that share the same ring, only one stope is valid to mine.

(5) $$\begin{array}{c} x_{iot}-\underset{\;r\in R(i)}{\sum }\;\underset{o\in O}{\sum }\;\underset{d\in D}{\sum }\;y_{rodt}\leq 0\;\forall \;i\in I,\;t\in T \end{array}$$

(6) $$\begin{array}{c} \underset{i\in I(r)}{\sum }\;\underset{o\in O}{\sum }\;x_{iot}-\underset{\;o\in O}{\sum }\;\underset{d\in D}{\sum }\;y_{rodt}\geq 0\;\forall \;r\in R,\;t\in T \end{array}$$

A similar pattern is formulated in equations (10)–(14), in which, if a candidate stope *i* is valid to be mined, the other stopes belonging to any of the sets described in Figure 2 are invalidated. Thus, the sum of both cannot exceed 1.

The non-overlapping constraint in equation (10) ensures that only one stope can be produced from all stopes sharing at least one block across all periods, where *i* represents the evaluated stope and $i^{\prime }$ represents a stope that belongs to the overlapping set $b_{i}$ .

(7) $$\begin{array}{c} \underset{o\in O}{\sum }\;\underset{t\in T}{\sum }\;x_{iot}+\underset{i^{\prime }\in b_{i}}{\sum }\;\underset{o\in O}{\sum }\;\underset{t\in T}{\sum }\;x_{i^{\prime }ot}\leq 1\;\forall \;i\in I \end{array}$$

The extraction level constraint in equation (11) ensures that practical drawpoint levels are established. Adjacent stopes that do not share the same extraction level ( $i^{\prime }\in e_{i}$ ) are not allowed to be mined across all time periods.

(8) $$\begin{array}{c} \underset{o\in O}{\sum }\;\underset{t\in T}{\sum }\;x_{iot}+\underset{i^{\prime }\in e_{i}}{\sum }\;\underset{o\in O}{\sum }\;\underset{t\in T}{\sum }\;x_{i^{\prime }ot}\leq 1\;\forall \;i\in I \end{array}$$

The adjacency constraint formulated in equation (12) guarantees that adjacent stopes cannot be produced in the same period to avoid creating large openings resulting in stability issues. As seen in this equation, candidate stope *i* is not produced simultaneously with its adjacent stopes set, $i^{\prime }\in j_{i}$ . An adjacent stope can be produced after the stope under consideration is backfilled and waited for the curing period.

(9) $$\begin{array}{c} \underset{o\in O}{\sum }\;x_{iot}+\underset{i^{\prime }\in j_{i}}{\sum }\;\underset{o\in O}{\sum }\;x_{i^{\prime }ot}\leq 1\;\forall \;i\in I,t\in T \end{array}$$

**Figure 6.**
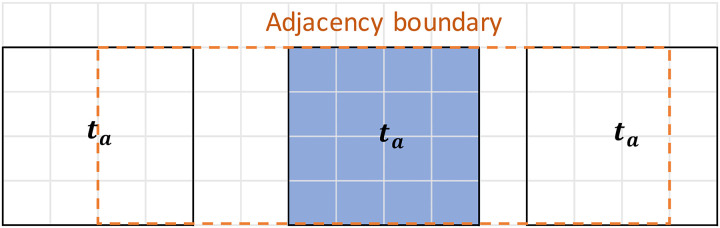
Illustration of adjacency constraint.

If there are blocks between two possible stopes, they may not be considered adjacent. When these blocks between stopes meet the minimum dimensionality requirement, these blocks form a pillar. Therefore, this stope is not subjected to adjacency constraint (Villaescusa, 2014). As seen in Figure 6, the blue stope is a candidate to be mined in period $t_{a}$ , and its adjacent stopes are framed in orange and located in a direct adjacency boundary. In a different scenario, the pillars between the blue stope and the stopes framed in black exist. If the pillar sizes meet pre-specified sizes given by the geotechnical engineer, it is not considered a non-adjacent stope. Equation (13) formulates this logic in the optimisation model.

Equation (13) ensures that the dimension of the rib and sill pillars is the width and height of the stope, with the largest dimensions among the closest stopes. Thus, for a targeted stope *i*, the set of overlapping stopes $\left(i^{\prime \prime }\in b_{i^{\prime }}\right)$ of any of its adjacent stopes $\left(i^{\prime }\in j_{i}\right)$ is invalidated for scheduling.

(10) $$\begin{array}{c} \underset{o\in O}{\sum }\;\underset{t\in T}{\sum }\;x_{iot}+\underset{i^{\prime \prime }\in b_{i^{\prime }}}{\sum }\;\underset{o\in O}{\sum }\;\underset{t\in T}{\sum }\;x_{i^{\prime \prime }ot}\leq 1\;\forall \;i\in I,i^{\prime }\in j_{i} \end{array}$$

The vertical offset constraint given in equation (14) ensures that stopes which lie directly above one another $\left(i^{\prime }\in o_{i}\right)$ are not mined at the same period. This prevents vertical planes of weakness from transversal across multiple extraction levels. If allowed to exist, they could encourage the failure of backfill material.

(11) $$\begin{array}{c} \underset{o\in O}{\sum }\;x_{iot}+\underset{i^{\prime }\in o_{i}}{\sum }\;\underset{o\in O}{\sum }\;x_{i^{\prime }ot}\leq 1\;\forall \;i\in I,t\in T \end{array}$$

The dimensions of pillars are a function of the ground conditions. The strategy of leaving permanent (sill and rib) pillars is critical when the appropriate distances between pillars and their dimensions are defined (Villaescusa, 2014). The permanent pillar constraint given in equation (15) delimits the allowable dimensions of the opening along the three directions ( $ext_{i}$ in *x*, *y*, and *z*). This constraint ensures that the width, length, and height of the total opening containing consecutive stopes does not exceed the maximum allowable opening extension $ext_{max}$ .

(12) $$\begin{array}{c} \underset{o\in O}{\sum }\;\underset{t\in T}{\sum }\;x_{iot}ext_{i}+\underset{i^{\prime }\in o_{i}}{\sum }\;\underset{o\in O}{\sum }\;\underset{t\in T}{\sum }\;x_{i^{\prime }ot}\;\;ext_{i^{\prime }}\;\;\leq ext_{max}\;\forall \;i\in I \end{array}$$

The mining capacity constraint given in equation (16) restricts mine extraction to not exceed the fleet capacities.

(13) $$\begin{array}{c} \underset{i\in I}{\sum }\;\underset{o\in O}{\sum }\;x_{iot}ton_{i}\leq M^{max}\;\forall \;t\in T \end{array}$$

Grade blending constraint, equation (17), ensures that the contained metal tonnage is between an upper and lower limit in any time period that a stope is in extraction. These constraints control grade variations fed into the plant for its efficient operation. This is evaluated for all the geological scenarios.

(14) $$\begin{array}{c} \underset{o\in O}{\sum }\;\underset{i\in I}{\sum }\;\underset{r\in R(i)}{\sum }\;\underset{d\in D}{\sum }\;g_{rs}\;y_{rodt}-\delta_{ts}^{g+}\leq G^{max}\;\forall \;\;t\in T,s\in S \end{array}$$

(15) $$\begin{array}{c} \underset{o\in O}{\sum }\;\underset{i\in I}{\sum }\;\underset{r\in R(i)}{\sum }\;\underset{d\in D}{\sum }\;g_{rs}\;y_{rodt}+\delta_{ts}^{g-}\geq G^{min}\;\forall \;t\in T,s\in S \end{array}$$

## Case study

**Figure 7.**
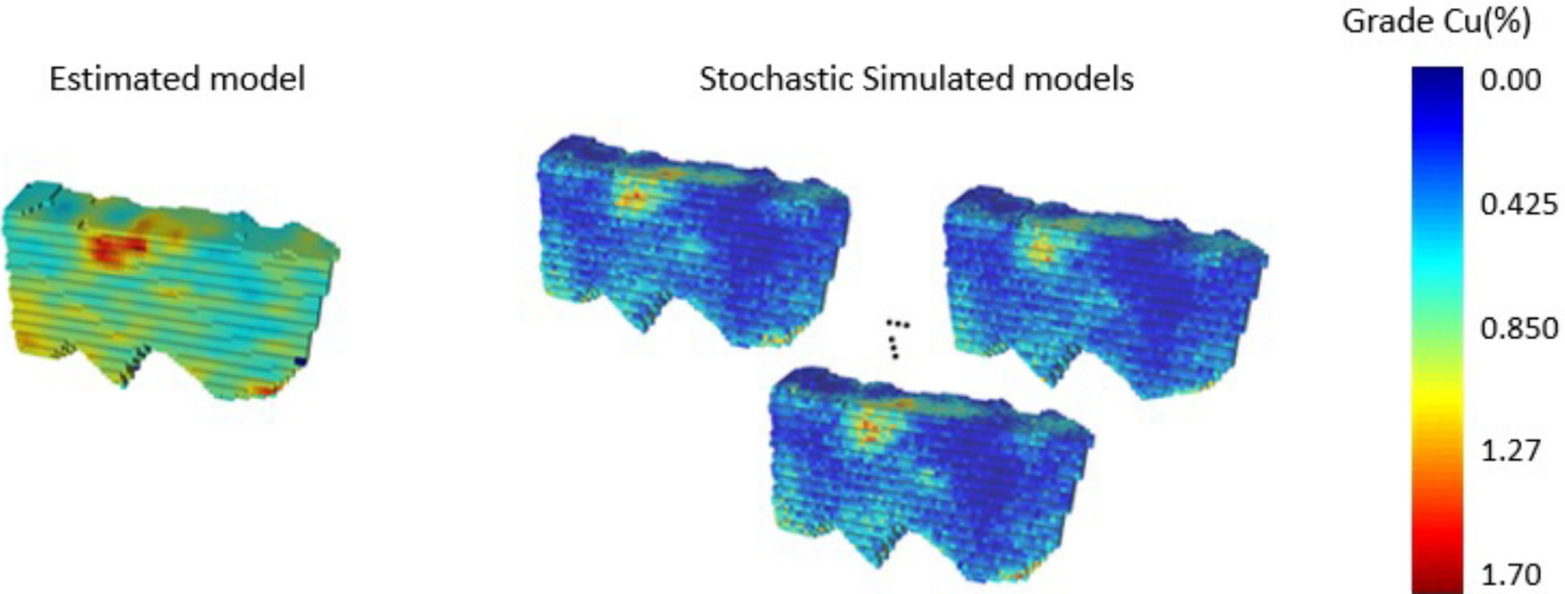
Estimated (ordinary kriging) and simulated (direct block simulation method (DBSim)) orebody models of the copper deposit of study.

A case study at a copper deposit was carried out to demonstrate the proposed approach. The mining method is sub-level stopping. The network starts with decline access. Orebody models were generated using geostatistical simulations and ordinary kriging (Goovaerts, 1997). Simulated models are based on the direct block simulation method (DBSim) (Boucher and Dimitrakopoulos, 2009; Godoy, 2002). The estimated and simulated block models provide input for the SIP model proposed in this paper. DBSim-based models are the major input for the SIP model, and the ordinary kriging-based model is the input for the MIP model and is used for benchmarking. Ten simulations were generated, each comprising 129,909 blocks with a size of 5 × 5 × 5 m. Another 10 simulations were generated for the risk analysis process of both the SIP and MIP approaches. Ten realisations of a deposit will provide stable results in the related stochastic optimisation process (Albor and Dimitrakopoulos, 2009; Montiel and Dimitrakopoulos, 2017). More realisations would generate more accurate results, but the size of the problem would significantly increase. Figure 7 shows the estimated and three realisations of the simulated model of the copper deposit.

### Decline options

A set of seven decline options is designed with considerations for 10% linear advancement and 5% curves with a radius of curvature of 30 m. The optimisation model will select one of these options as the main access to mine. As discussed, the decline location significantly affects production direction, stope layout, and sequencing due to varying materials handling costs. Two important factors were considered in defining the decline positions: Geological conditions, water presence, and geomechanical operability. It was assumed that these options were provided by the geotechnical team of the operation, considering rock characteristics, lithology, geology, and water conditions. The materials handling costs of each stope are different according to each decline option. Therefore, stope layout and production scheduling will differ for each decline option. A safety shield is respected for all decline options regarding the required safety distance from the orebody.

**Figure 8.**
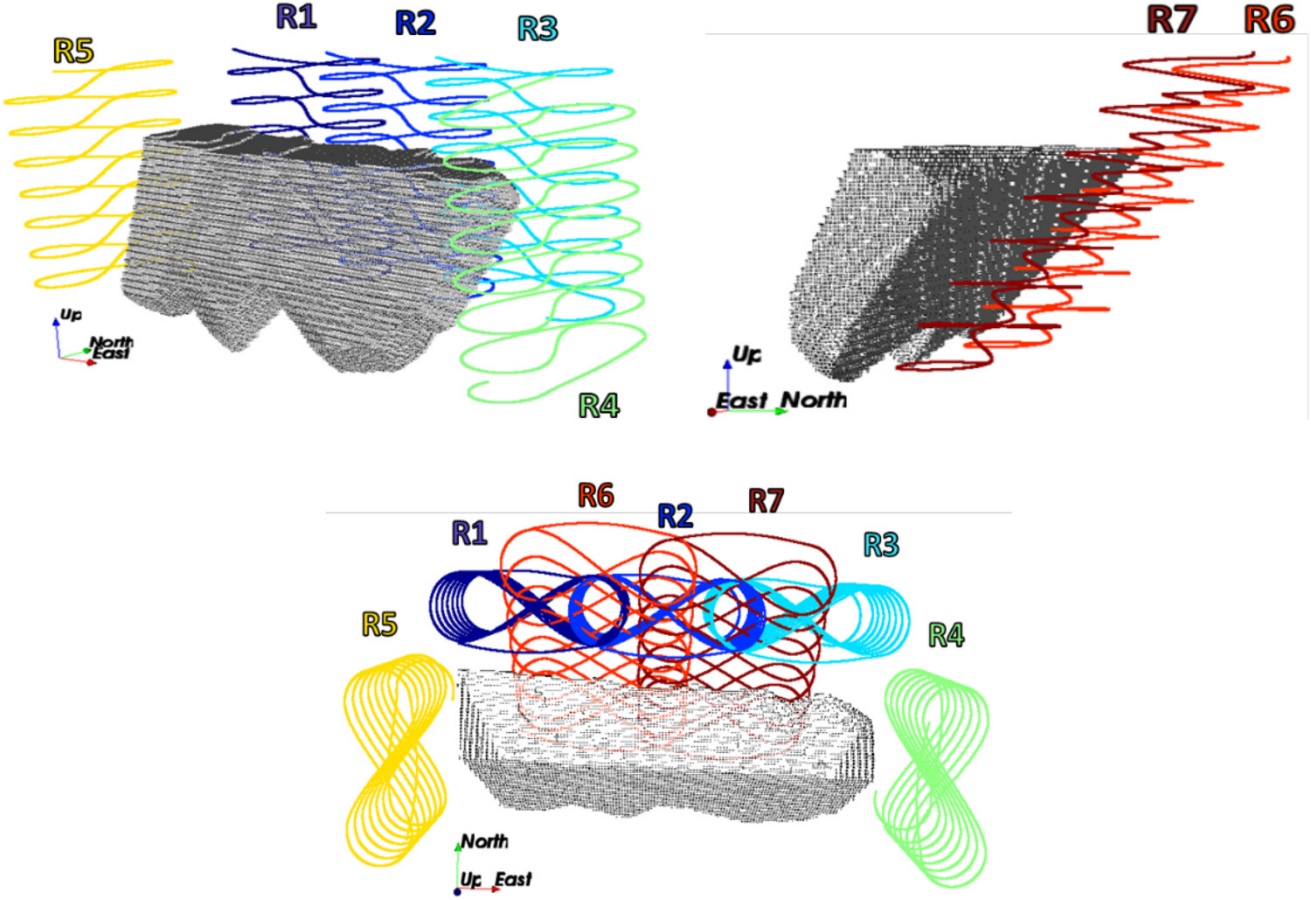
The location of decline options provided by the geotechnical team in the case study in three views.

Figure 8 shows the seven decline options in three different views of the orebody. The optimisation models aim to select the decline that minimises the capital cost of declined construction and the operating cost of materials handling realised through this decline. On the other hand, the revenue obtained from the extraction of a stope also affects the optimisation process. The model formulation focuses on maximising revenue and minimising mining costs, including materials handling. The selected decline should be close to high-grade zones. However, since there are a large number of possible stopes in the model, a decision-maker should rely on an optimisation engine. Declines options R1, R2, R3, R6, and R7 are located on the footwall of the orebody, where R1, R2, and R3 are designed vertically, and R6 and R7 follow the dip of the deposit. Decline options R4 and R5 are the two corner cases designed vertically. The seven decline options cover most of the possible connections that can be obtained for decline-stope access networks.

### Operational considerations

Annual production is planned as 486,000 tonnes with a metal production between 4374 and 5832 copper tonnes. The life-of-mine is found by the optimiser, where the output corresponds to the scheduled stope layout. The stope sizes, $\left(b_{i}\right)$ , are 20 m for the *x*-axis, 30 m for the *z*-axis and for deposit geometry, a single size along the *y*-axis is defined in 50 m (corresponding to 10 rings).

Table 1 summarises the operational, economic, and risk control parameters used for the optimisation process applied to both MIP and SIP cases.

**Table 1.** Operational, economic, and risk-controlling parameters.

Parameters	Unit	Value
**Blocks**		
- Dimensions	metres	5 × 5 × 5
- Number of blocks		16,225
- Block tonnage	tonnes	337.5
**Rings**		
- Dimensions	metres	20 × 5 × 30
- Number of rings		97,812
- Ring tonnage	tonnes	8100
**Stopes**		
- Dimensions	metres	20 × 50 × 30
- Number of stopes (SIP)		19,998
- Number of stopes (MIP)		16,339
- Stope tonnage	tonnes	81,000
**Stope mining recovery facto**r	%	93
**Copper metal price**	$/tonnes	8500
**Economic discount rate**	%	10
**Geological discount rate**	%	10
**Mining cost**	$/tonnes	20
**Processing cost**	$/tonnes	13
**Preparation cost**	$/m	1200
**Development cost**	$/m	1800
**Transportation cost**	$/Tn.m	0.004
**Number of destinations**		2
**Number of simulations**		10
- For the SIP		10
- For the risk analysis		
**Number of decline options**		7

The mill feed head grade should be between 0.90% and 1.20% Cu per period. The geological risk discount rate, which can control the risk over time, was also included, showing that for a high rate, the SIP will place more emphasis on a less risky schedule for the early periods, resulting in a decrease in the risk of not achieving the planned target and ensuring that deviations from the production targets are minimised. An initial optimisation process with zero penalty costs was tested, resulting in a mine plan that deviated from the lower production targets. Therefore, other penalties were tested, resulting in a penalty cost of 100$ needed to avoid violating the production targets along the life-of-mine.

Rings can be classified as ore and waste, but stopes should only be mined if their total value generates profit. Otherwise, it would not make sense to invest in accessing and extracting waste material, since the present sublevel method does not need precedence between stopes, it only needs accessing routes. In this sense, a reduction in the number of binary variables (such as $x_{iot}$ and in consequence $y_{rodt}$ ) will significantly reduce the solution time. The size reduction strategy implemented in Leite and Dimitrakopoulos (2014) for a set of simulated block models proved not to alter the optimality of the solution, so it is adopted here by defining a probability threshold for the classification of stopes into ore and waste. The total number of possible stopes for the SIP model was 443,568, being reduced to 19,998 stopes, while for the MIP model, since only an estimated block model is used, the economic evaluation was directly done for each unique stope value, reducing the number of stopes to 16,339. The difference in the number of stopes between the MIP and SIP explains the smoothing effect of the kriging methods, misrepresenting extreme values and not reproducing patterns of spatial continuity that provide an assessment of spatial uncertainty (Goovaerts, 1997; Journel and Huijbregts, 2004). Table 2 provides the parameter values and units used for capacity, grade target range, and dollar penalty costs from target deviations.

**Table 2.** Capacity, targets, and penalties parameters.

Parameters	Unit	Value
Mining capacity	tonnes	486,000
Grade targets	Cu%	0.90–1.20
Penalty costs - Upper deviation from grade targets - Lower deviation from grade targets	$ per unit deviation	0 100

### Performance analysis

The MIP and SIP models were solved with CPLEX 12.8 (ILOG) optimiser, using an i7-8700 computer with 32.0 GB RAM on a 64-bit operating system. In the initial run, the total number of decision variables was 1,483,741 for the MIP and 1,509,354 for the SIP, while the number of constraints was 2,450,360 for the MIP and 2,800,462 for the SIP. The problem size was reduced by a preprocessing step. The stope sets processing step took 56 s, while the optimisation of the SIP model required ∼10 h and the MIP model required ∼4 h.

**Figure 9.**
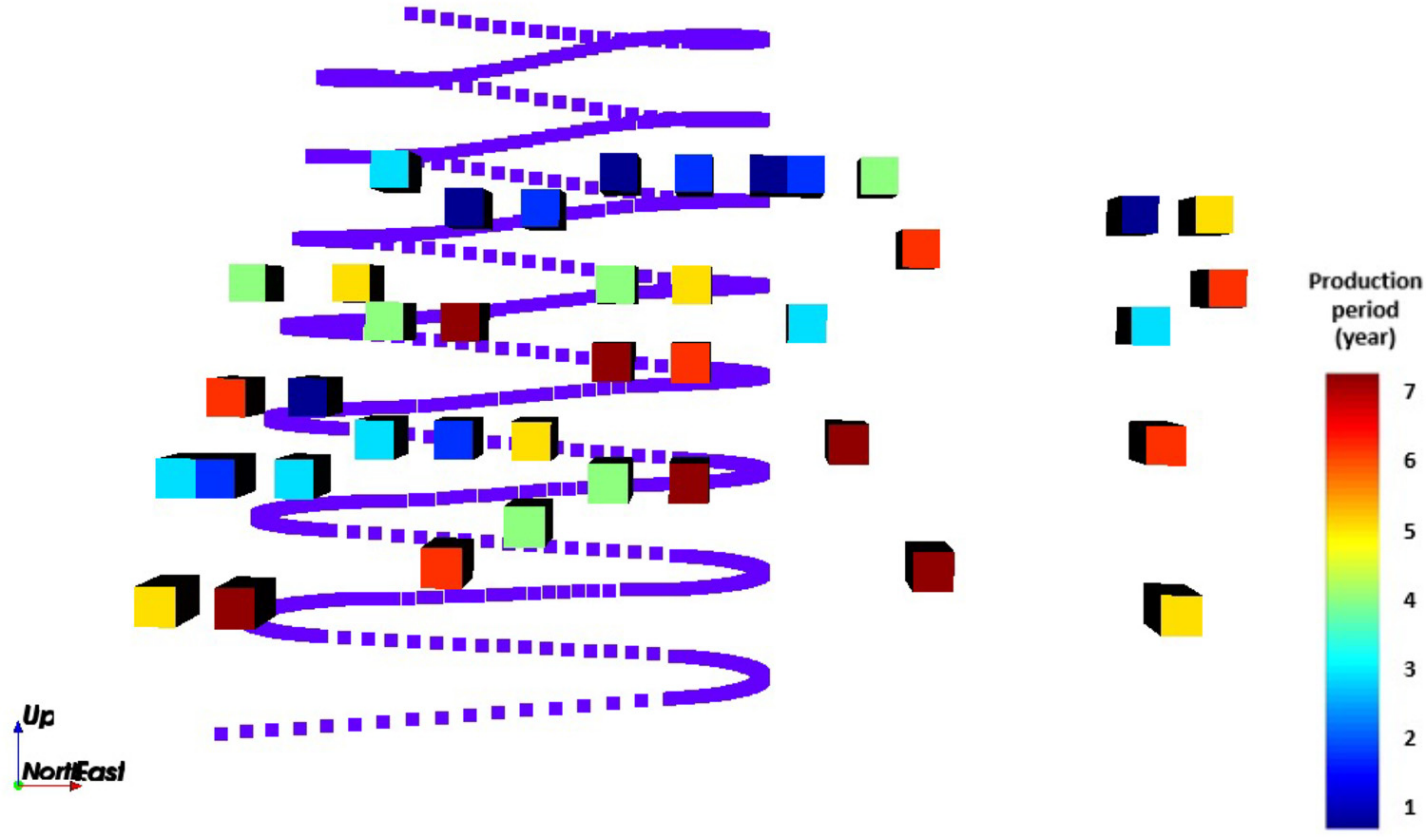
Front view of the optimised schedule obtained by the stochastic integer programming (SIP) model (scheduled stopes to produce, production periods, and the selected decline).

**Figure 10.**
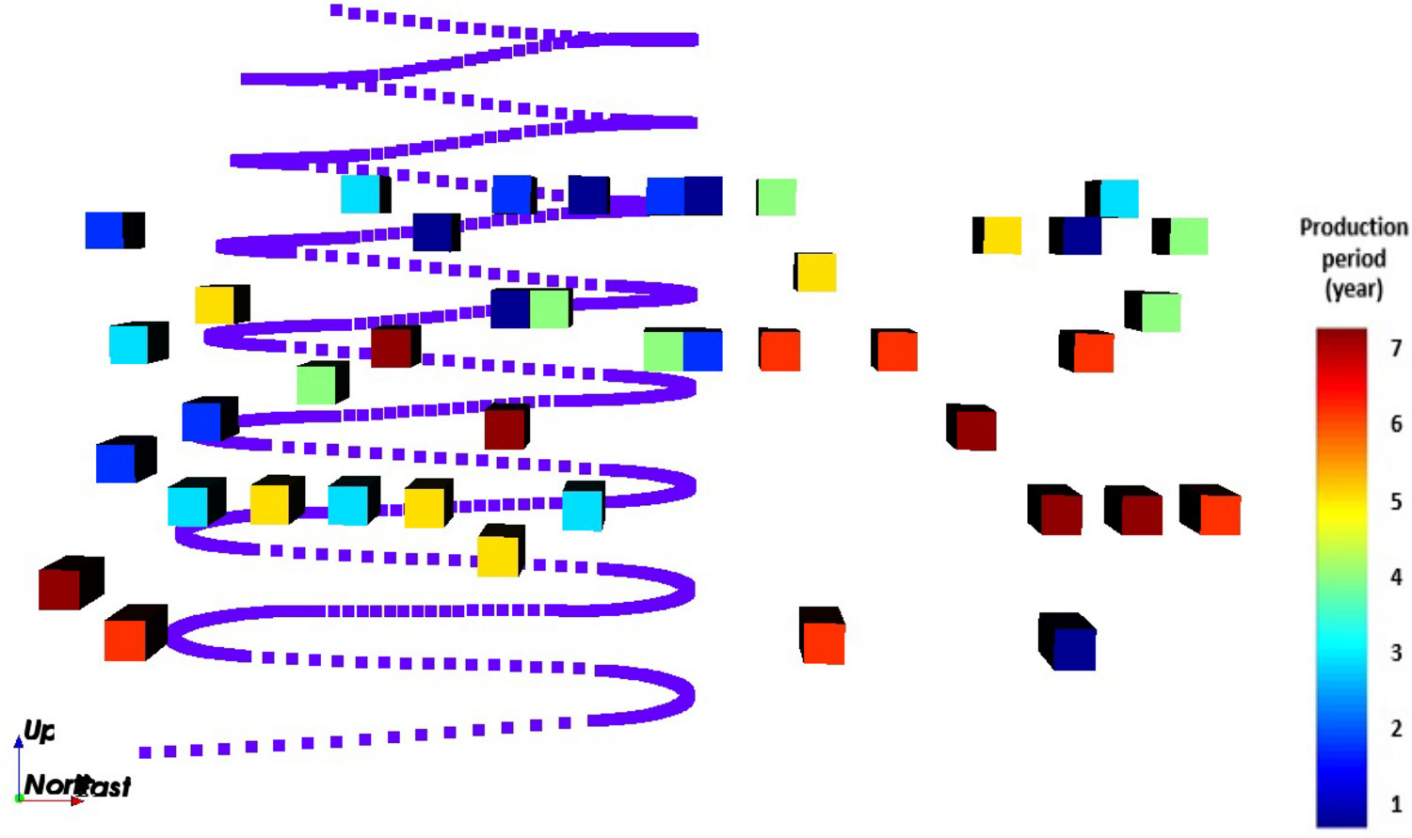
Front view of the optimised schedule obtained by the mixed integer programming (MIP) model (scheduled stopes to produce, production periods, and the selected decline).

When comparing the schedules obtained with the SIP (Figure 9) and the MIP (Figure 10), the models select the same decline (R6) and the same life-of-mine. However, as can be seen from the figures, the stope layout and sequence are different. This difference is due to the geological grade variation in rings and stopes. The SIP model seeks a trade-off between NPV and the target deviations given in the mathematical formulation.

**Figure 11.**
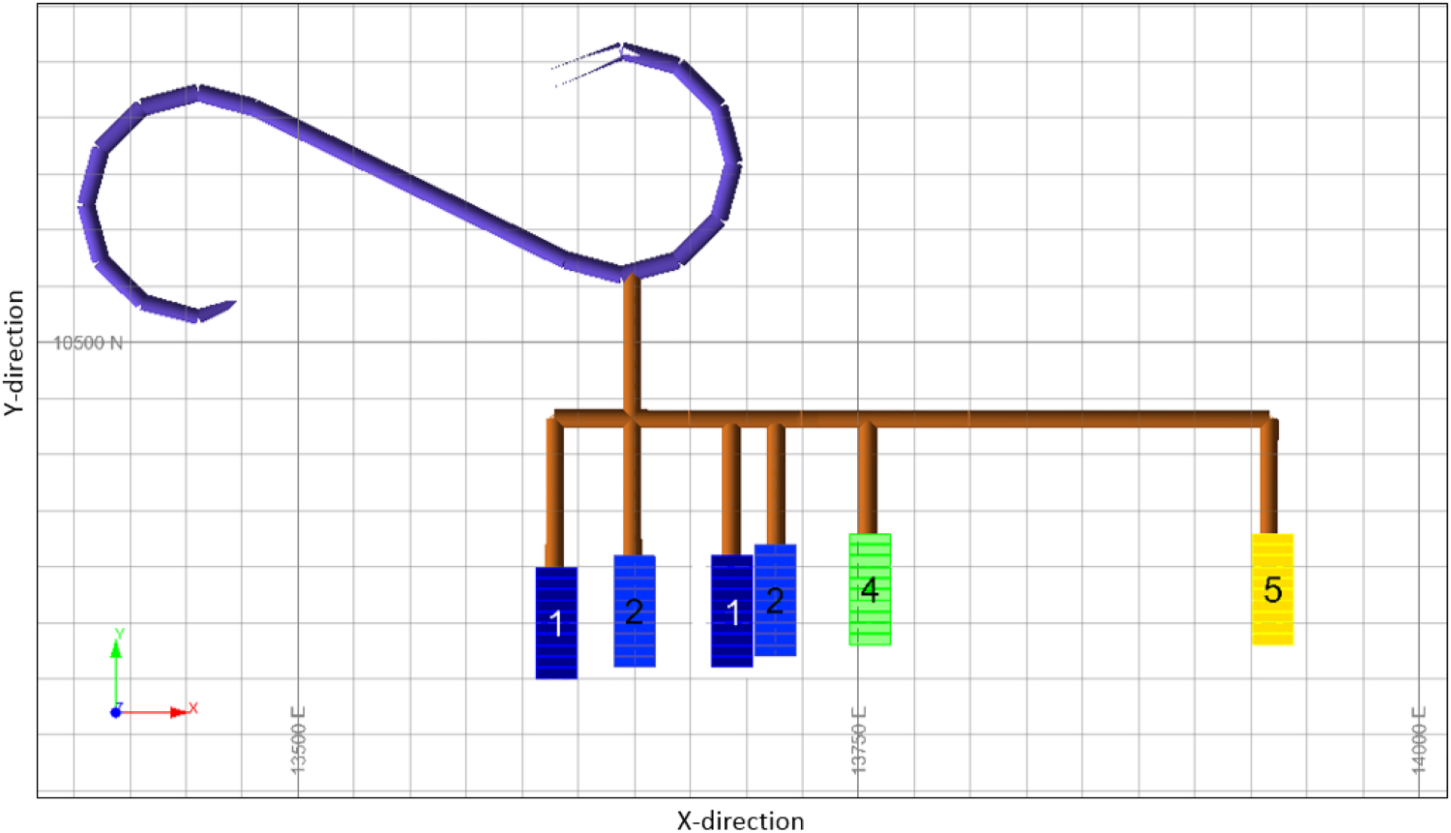
Plan view of a sector of stopes selected for production, decline, and drifts.

The optimisation model pushes waste blocks to include pillars and ore blocks to include production stopes. Thus, internal dilution is minimised. The only source of internal dilution is the waste rings that must inevitably be included in stopes. The plan view at one level of the scheduled stopes with the SIP model is shown in Figure 11. As can be seen from this figure, the numbers within each stope represent its scheduled production period on a level. For example, the stopes shown in midnight blue (Number 1) will be produced in the first period, the stopes shown in azure blue (Number 2) will be produced in the second period, and so on. This figure further shows that the stopes close to decline are produced in earlier periods under geomechanical constraints. The section shows the decline option selected by the optimiser (decline 6). The access drifts are visualised as connecting to the stopes, shown in brown. The figure shows the stopes that are accessible from a level of the decline. They do not overlap. There is no simultaneous production of adjacent stopes, maximum lengths and widths are respected, and pillars are considered between stopes.

**Figure 12.**
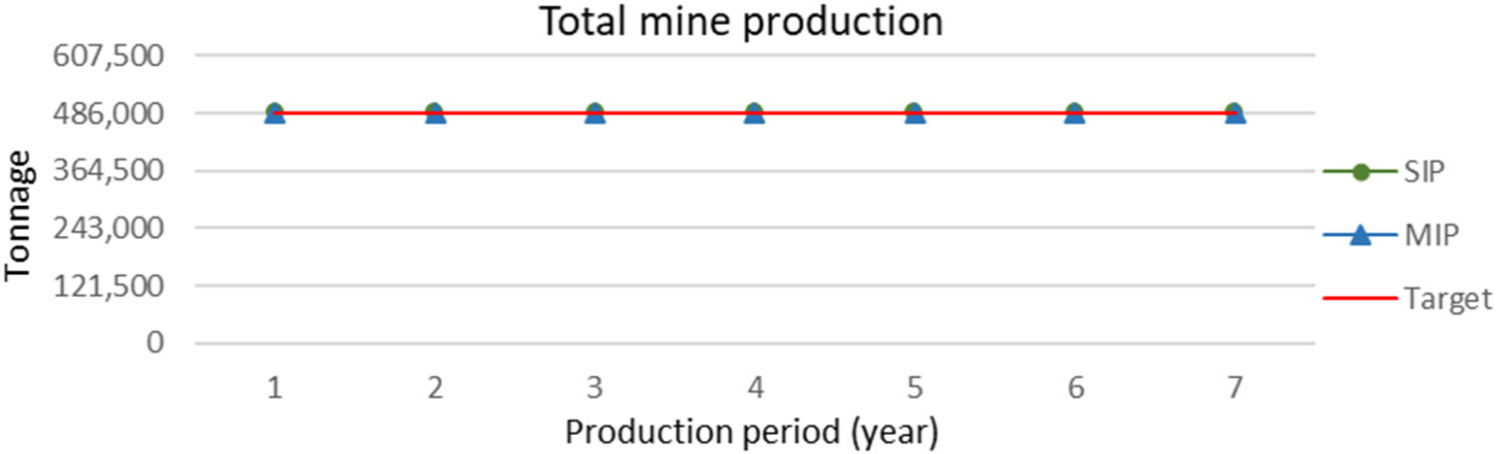
Yearly mine production (tonnes) for the SIP and MIP models.

SIP: stochastic integer programming; MIP: mixed integer programming.

Figure 12 shows that the production in each period is equal for both MIP and SIP, having a constant production of 486,000 per year, which is the maximum mining and processing capacity. Both models produce a 7-year schedule with the same cumulative production of 3,402,000 tonnes. The figure shows the overlapping curves of yearly production for the SIP and the MIP that meet the target.

**Figure 13.**
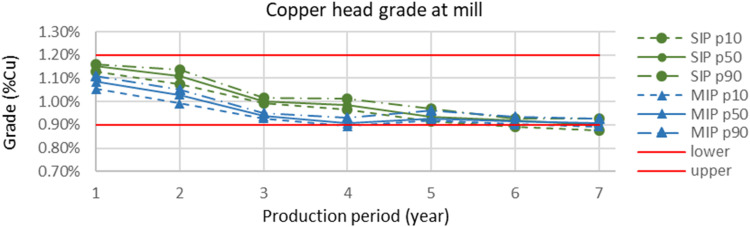
Yearly copper head grade (Cu%) at the mineral processing for the SIP and MIP models along with confidence intervals.

SIP: stochastic integer programming; MIP: mixed integer programming.

Figure 13 shows the average grade of the ore sent to mineral processing in each period for MIP and SIP models, along with their confidence intervals. Two red lines denote upper and lower grade limits. In the first four years, the average grades of ore sent to mineral processing by SIP (green lines) are higher than that of the MIP model (blue lines). Towards the end of mine life, MIP generates higher ore grades. Also, a small deviation from the grade requirement was observed at the end of my life. Given that the SIP model allows deviations at a cost, this deviation is acceptable. This issue can be eliminated if higher penalty values are used for deviations.

**Figure 14.**
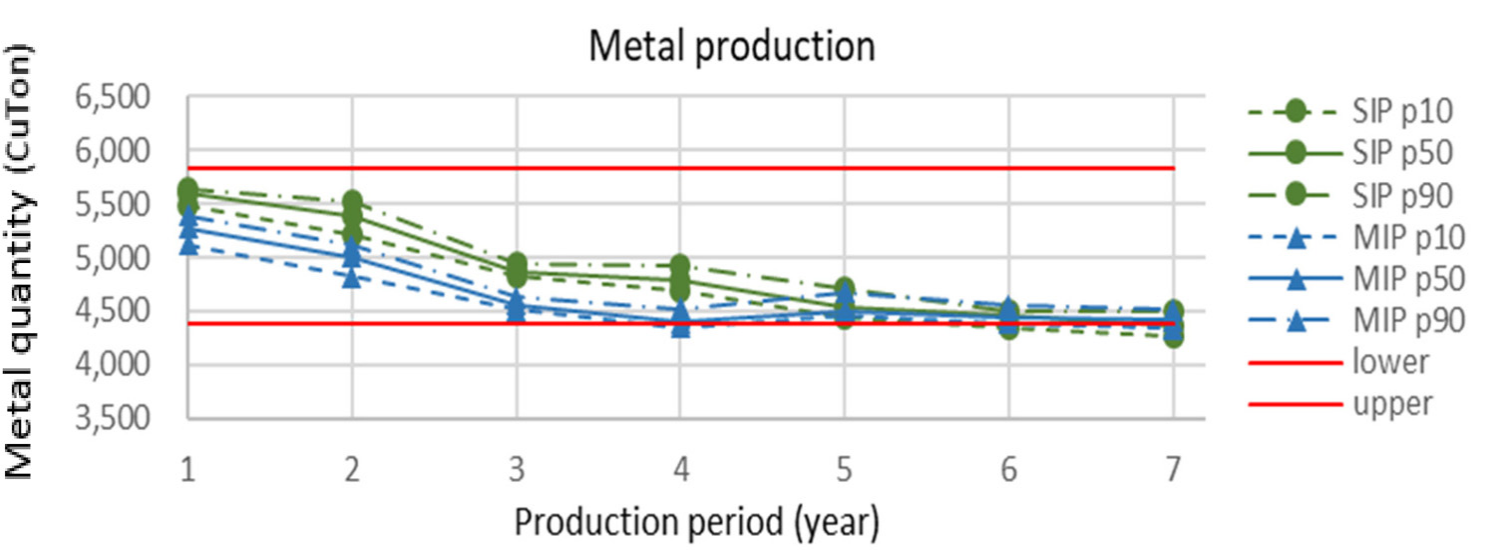
Yearly metal production (Cu tonnes) for the SIP and MIP models along with confidence intervals.

SIP: stochastic integer programming; MIP: mixed integer programming

A similar pattern, which is seen for the head grades of mineral processing, is also observed for metal quantity in Figure 14. The dashed lines show confidence intervals. The straight red lines denote upper and lower grade limits. As seen in this figure, the year-to-year metal quantities scheduled by SIP are more than those of MIP for the first five years of mine life. The exact amount of ore is produced for each period of SIP and MIP models, so observing a similar pattern for grades and metal quantities is consistent.

**Figure 15.**
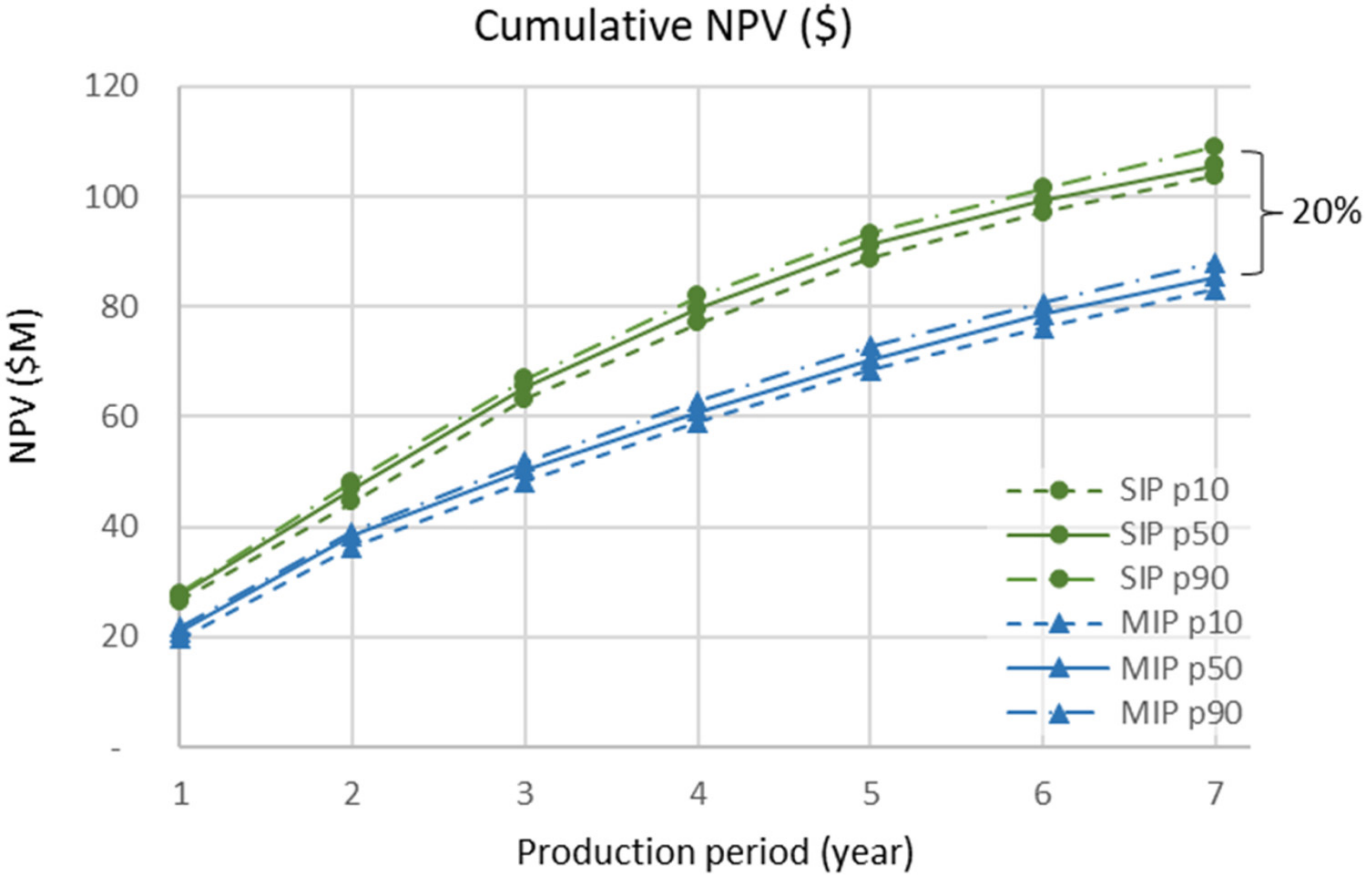
Cumulative NPVs that the project generates for SIP and MIP models along with confidence intervals.

NPV: net present value; SIP: stochastic integer programming; MIP: mixed integer programming.

Figure 15 shows cumulative NPVs generated by SIP and MIP, along with their confidence intervals. As can be seen, SIP outperforms MIP. The difference between NPVs also grows in later years of operation. In total, SIP generated 20% more NPV. In the early years of operation, the NPV difference grew; however, after five years, it remained constant. Given that the metal quantities to be produced are similar in the later years, as seen in Figure 14, the outcomes of Figure 15 are consistent.

The optimisation model formulated in this paper aims to maximise NPV. Geomechanics control is incorporated by equations (12) to (15). It is arguable whether these equations may sufficiently control stability risks and unplanned dilution. Therefore, the production schedule generated in this research should be subjected to stability assessment work, such as the stability graph method and others applicable. Necessary modifications should be made if needed.

Also, decline options could be more complicated, considering the capacities of materials handling systems and geomechanics constraints. However, to keep the generality and the discrete nature of the problem, decline options consider turning radius and gradient constraints only. The selected decline option should be subjected to the feasibility test from a geomechanical viewpoint. If needed, the necessary adjustment should be implemented.

## Conclusions

This research proposed a joint optimisation method for SLS UMP. The formulation based on a two-stage SIP jointly optimises the stope layout, production scheduling, and access network. The objective is to maximise NPV and minimise planned dilution. In the SIP models, deviations from production targets are allowed at the expense of a penalty cost. Given the uncertain grade, deviations from production targets are inevitable during operation. Therefore, an outcome deviating from the targets is acceptable and even more representative.

This approach provides a dynamic stope size controlled by the minimum and maximum number of blasting rings delimited by geomechanical and operational conditions. The SIP approach presented integrates the three main components of the mine plan and considers the interaction between them, guaranteeing the optimality of the solution. Constraints are needed to avoid prohibited designs, such as overlapping stopes and continuous stopes at different extraction levels, and to control geomechanical aspects, such as temporary and permanent pillars (controlled by the adjacent and offset stope sets).

The approach was applied to a copper porphyry deposit, where the SIP and its MIP equivalent are compared to show the value of the stochastic solution. The MIP model used an estimated model generated with the ordinary kriging method, and the SIP used a set of 10 stochastic simulations generated with the DBSim method. Another set of 10 simulations was generated for the risk analysis. The importance of including uncertainty is demonstrated, where the stochastic or risk-integrating approach defines a schedule that improves the NPV over life-of-mine by 20%, compared with the MIP model.

The proposed model can be extended to different extraction directions. Currently, the model is limited to the rings along the *y*-axis. The proposed model employs a certain number of declines. This model can also be furthered by adding possible decline and shaft accesses. The current work accepts the declines as a priory option. The optimisation model can be extended to include multiple processing streams. Finally, it should be noted that geomechanical constraints are paramount. The proposed model may interact with stability assessment software to verify if the production schedule is feasible from a geomechanical viewpoint. If not, the model parameters may be altered until an optimal plan that is feasible from the stability point of view is identified. In the proposed model, the problem is formulated with respect to the information given by the geotechnical team.

## Appendix

### Notation

**Indices**
d: destination; d=0,1 , where d=0 is for material sent to waste and d=1 for the mill.
i: stope; i=1,…,I
o: decline option; o=1,…,O
r: ring; r=1,…,R
s: simulation; s=1,…,S
t: period; t=1,…,T

**Parameters**
*ac*: preparation advancement cost per meter ($/m)
*c^g±^*: upper and lower target deviation costs for metal production ($)
$c_{t}^{g+},c_{t}^{g-}$: costs for deviating the upper and lower bounds of metal content targets in period *t* ($) (16) $$\begin{array}{c} c_{t}^{g\pm }=\frac{1}{{\left(1+dr^{geo}\right)}^{t}}\;c^{g\pm } \end{array}$$
*dist_io_*: preparation distance from decline option *o* to stope *i* (m)
*dist_rod_*: distance that ring *r* is transported to destination *d* for decline option *o* (m)
*dr^econ^*: economical discount rate, $dr^{econ}\in [0,1]$
*dr^geo^*: geological discount rate, $dr^{geo}\in [0,1]$
*ext_i_*: extensions of stope *i* in coordinates *x*, *y*, and *z*
*ext_max_*: maximum allowable opening dimension for *x*, *y*, and *z*
*f*: development cost per meter ($/m)
*G^min^* and *G^max^*: min and max head metal grade allowed at the mill
*g_rs_*: ore grade (%) of ring *r* in scenario *s*
*l_o_*: length of decline option o
*mc*: mining cost per tonne ($/Tn)
*M^max^*: mining capacity
*pc_d_*: processing cost per tonne ($/Tn)
*rec_d_*: metallurgical recovery (%) of metal, $rec_{d}\;\in [0,1]$
*tc*: transportation cost ($/Tn.m)
*ton_i_*: tonnage in stope *i*
$\propto_{rodts}$: discounted cash flow obtained from ring *r* in period *t*, scenario *s*, and decline option *o* (17) $$\begin{array}{c} \propto_{rodts}=\frac{1}{{\left(1+dr^{econ}\right)}^{t}}\;[\left(ton_{r}\;g_{rs}\;rec_{d}\;\pi )-ton_{r}(pc_{d}+dist_{rod}\;tc\right)] \end{array}$$

$\beta_{iot}$: mining and preparation costs for mining stope *i* using decline option o (18) $$\begin{array}{c} \beta_{iot}=\frac{1}{{\left(1+dr^{econ}\right)}^{t}}\;(ton_{i}\;mc+dist_{io}\;ac) \end{array}$$
π: selling price of the element mined
